# Practice-based skill acquisition of pushrim-activated power-assisted wheelchair propulsion versus regular handrim propulsion in novices

**DOI:** 10.1186/s12984-018-0397-4

**Published:** 2018-06-26

**Authors:** Rick de Klerk, Thijs Lutjeboer, Riemer J. K. Vegter, Lucas H. V. van der Woude

**Affiliations:** 1 Center for Human Movement Sciences, University Medical Center Groningen, University of Groningen, Groningen, The Netherlands; 2 Center for Rehabilitation, University Medical Center Groningen, University of Groningen, Groningen, The Netherlands

**Keywords:** Cyclic exercise, Ergonomics, Mechanical efficiency, Motor learning

## Abstract

**Background:**

Regular handrim wheelchair (RHW) propulsion is straining for the upper extremities and wheelchair users often experience overuse problems. A recent advancement in wheelchair technology that could assist users is the pushrim-activated power-assisted wheelchair (PAPAW). PAPAWs are challenging to control, yet it is unclear how people learn to use a PAPAW. The purpose of this study is to examine early skill acquisition through practice in PAPAWs and compare it with RHWs.

**Methods:**

Twenty-four able-bodied novices were randomly allocated to either the RHW group or the PAPAW group. The experiment consisted of five sessions with three blocks of 4 min steady-state practice at 1.11 m/s and 0.21 W/kg. Finally, a transfer to the other mode was made. Data were collected with a drag-test, breath-by-breath spirometry, and a motion capture system. The last minute of each four-minute block was used for analysis. A mixed analysis of variance (ANOVA) was used to test for group, time, and interaction effects.

**Results:**

Both groups improved their (assisted) mechanical efficiency, reduced their stroke rate, right-left and forward-backward deviation on the treadmill, and had a lower rate of perceived exertion (RPE) over time. (Assisted) mechanical efficiency was higher for the PAPAW group than for the RHW group and RPE was lower. However, left-right and forward-backward deviation was also found to be higher in the PAPAW group.

**Conclusions:**

At the group level the energetic cost of RHW and PAPAW propulsion can be lowered through low-intensity practice in novice users. The PAPAW is more ‘efficient’ than the RHW given the reduced energy requirement of the user from the motor assist, but more difficult to control. Future studies on PAPAWs should focus on the control needs of the user and their interaction with the power-assist technology.

## Background

A large section of persons with a disability is dependent on wheelchairs for locomotion with approximately 70 million people worldwide that rely on a wheelchair for their mobility [1]. Regular handrim wheelchairs (RHW) increase independent mobility for people with lower limb impairments, which improves their quality of life [2], while keeping them physically active to prevent a non-active lifestyle [3]. However, wheelchair propulsion is known to have a low mechanical efficiency and wheelchair users often experience overuse problems, especially in the shoulders, but also in the elbows and wrists [2, 4, 5].

Hence, alternatives to RHWs have been developed in the past for those with shoulder pain, limited arm function, or upper body capacity. One such alternative is the fully-powered wheelchair [6]. Although they might reduce strain on the shoulders and arms, they are expensive, require vans and lifts for transportation [7], and they encourage a much less physically active lifestyle [8]. Thus, various other substitutes to handrim and fully-powered wheelchairs have been proposed such as crank and lever propelled wheelchairs. Whereas these systems prove to be less physically straining, there are several practical limitations that prevent regular use [9].

A more recent advancement in wheelchair technology is the pushrim-activated power-assisted wheelchair (PAPAW). A PAPAW can be seen as a hybrid between fully-powered wheelchairs and RHWs. The aim of PAPAWs is to prevent overuse complications, while maintaining a level of physical activity [10], increasing the social participation and independence of the user. They are equipped with an integrated electric motor mounted in the wheels or wheelchair frame [10] that is activated by pushing the handrims, as you would if you were propelling a RHW [11]. Previous research has shown that using a PAPAW can reduce heart rate [11–13] and rate of perceived exertion [7, 11, 14], lower metabolic cost [13, 15, 16], decrease shoulder range of motion during propulsion [13, 17], and increase distance travelled throughout the day [18, 19] when compared with RHWs.

However, handrim wheelchair propulsion is a complex motor task in which the bimanually applied forces determine both the speed and direction of locomotion [20]. Hence, PAPAWs might be more challenging to control since small interlimb differences are amplified by the wheelchair motor assist system, causing changes in direction. Therefore, the motor skill of the user is thought to be important for the effectiveness of PAPAW use. Yet, no research is available that examined the process of skill acquisition of PAPAW propulsion during the initial stages of motor learning.

Wheelchair propulsion is a cyclic motor task, which makes it possible to evaluate performance using energy consumption as a generic outcome measure of submaximal steady-state motor performance [21–23]. Skilled individuals will require less internal energy to produce the same amount of external power, this ratio is called mechanical efficiency. In this paper, we use the term ‘assisted mechanical efficiency’ for PAPAWs, since the external power output contribution of the motor and user cannot be disentangled.

An increase in this efficiency because of practice has already been demonstrated on ski-simulators, rowing-ergometers and in RHWs [21, 23–26]. Vegter and colleagues showed that healthy novices starting to learn handrim propulsion under steady-state low-intensity practice improve both efficiency and propulsion technique after bouts of 12–80 min practice [21, 27]. These time associated changes through practice have not yet been studied in PAPAWs.

Another commonly used measure in motor learning studies is the amount of endpoint variability [28]. In wheelchair propulsion on a motor driven treadmill this can be translated to the ability to steer the wheelchair where it is required to be steered (in speed and position), akin to a tracing task. This will be referred to as control. Wheelchair propulsion on a motor driven treadmill provides a mechanically valid [29] and representative environment [25]. It also requires the occupant to pay close attention in order to stay on the track given the limited length and width of the belt. Therefore, a second measure for skill is proposed, which consists of the ability to maintain more or less a steady position on the treadmill. It is expected that someone with a higher level of skill will show less position-related variability. Furthermore, RHWs are expected to show less variability than PAPAWs, since small interlimb differences in force application are not amplified by the power-assist motors.

As wheelchair propulsion is novel to persons in early rehabilitation after losing their walking ability and to many able-bodied participants, it allows for the use of able-bodied participants as a model to study the early acquisition of wheelchair propulsion proficiency. Additionally, able-bodied participants form a less heterogeneous group, allowing the study of motor learning in absence of impairment [21].

The aim of the current study is to explore the initial skill acquisition through low-intensity practice of able-bodied participants in PAPAWs and compare them to able-bodied participants that performed the same protocol with RHWs. To that end both groups performed five practice sessions consisting of three blocks of 4 min steady-state practice at 1.11 m/s and 0.21 W/kg on a motorized treadmill. After the practice sessions, a crossover trial was performed to investigate the transfer from one mode to the other.

It was hypothesized that mechanical efficiency and control improves over time in both propulsion modes. It was also hypothesized that RHWs are easier to control but are more physiologically demanding. Moreover, a positive transfer of skill was expected between the two modes, since PAPAW propulsion is thought to be at least partly similar to RHW propulsion [30]. If the skills obtained during one mode (RHW or PAPAW) are not specific to that mode, a higher performance should be expected from someone that practiced in one mode than someone who has never used a wheelchair. However, if the skills obtained during practice are highly specific to a mode, a negative or zero transfer of skill could take place. Information on the transfer of skill between RHWs and PAPAWs could provide insight on the specificity of the mode.

## Methods

### Participants

**Fig. 1:**
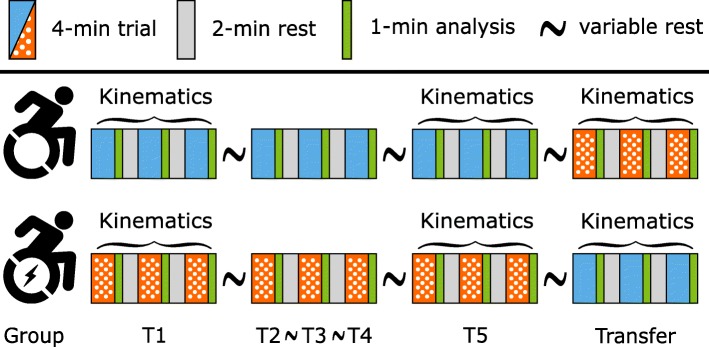
Participants were randomly assigned to one of two groups. One group practiced in a RHW (blue) and the other one in a PAPAW (orange/dotted). Participants practiced during five sessions, each consisting of three blocks of four minutes. The transfer took place after T5 and a 10-min rest. Physiological measurements were performed at each session and kinematics were only collected during the first (T1), final (T5) and Transfer session

A convenience sample of 17 able-bodied male and seven female university students was drawn for this randomized controlled trial. The participants had no prior experience in wheelchair propulsion and did not have any contraindications for exercise. Participants were randomly allocated into one of two groups (RHW and PAPAW, Fig. 1). Characteristics of both groups are given in Table 1. Participants were informed with an information letter about the research and provided written informed consent before taking part in the study. The study was approved by the Ethics Committee of the Center for Human Movement Sciences Groningen (ECB/2016.01.15_1), University Medical Center Groningen, University of Groningen, The Netherlands.

**Fig. 2:**
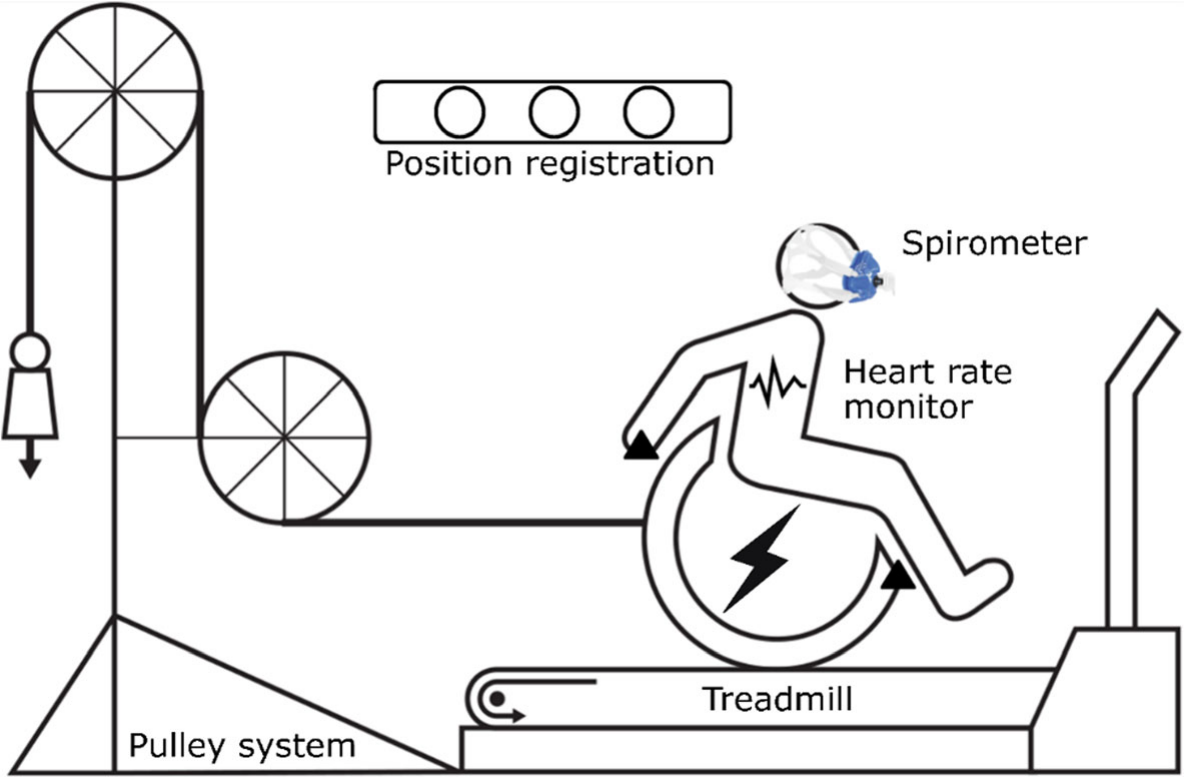
Experimental setup for all five sessions. The spirometer and heart rate monitor were used during every session. Position registration was only used during the first first, last, and transfer session. Treadmill speed was 1.11 m/s and power output was set at 0.21 W/kg with the pulley system. Black triangles exemplify the position of the two cluster markers. Modified figure from Vegter et al. [20]

**Table 1:** Participant characteristics (mean ± SD) per group

	RHW group (*n* = 12)	PAPAW group (*n* = 12)	*p*-value
Sex (m/f)	8/4	9/3	*p* = 1.000^a^
*Handedness (r/l)*	10/2	11/1	*p* = 1.000^a^
*Age (yrs)*	22 (±2.5)	22 (±3.3)	*p* = 0.725^b^
*Height (m)*	1.84 (±0.07)	1.82 (±0.08)	*p* = 0.699^b^
*Weight (kg)*	75.6 (±11.6)	77.4 (±11.6)	*p* = 0.709^b^

^a^2-sided p-value of a Fisher’s exact test. ^b^2-sided *p*-value of an independent samples t-test

### Protocol

All experiments were performed in a climate-controlled laboratory (20 degrees Celsius, 45% humidity) at the Center for Human Movement Sciences, University Medical Center Groningen, Groningen, The Netherlands on a level treadmill (Fig. 2) with a 2.0 m long by 1.2 m wide belt (Motek-Forcelink, Culemborg, The Netherlands). Each group practiced five times at a submaximal external power output of 0.21 W/kg bodyweight (Fig. 1). Each of the five practice sessions (T1-T5) lasted for 16 min with three blocks of 4 min of wheelchair propulsion (total training dose: 60 min) at 1.11 m/s (4 km/h) and two breaks of 2 min in between. A resting period of approximately 2 days separated the individual sessions. After the fifth session and 10 min of rest, the wheels were switched and the participants performed one additional session of equal length, the transfer session, in the other mode. Participants received no help or feedback; the only instruction was to stay in the center of the treadmill. Physiological measurements were performed during every session and participants were asked to rate their exertion after every session. Kinematics were only collected during T1, T5, and the transfer session. Data were collected during each practice block and the fourth minute of each block was used for analysis to ensure steady-state conditions.

### Wheelchair

The power-assisted wheels used in the current study for the PAPAW mode were the commercially available 24-in. Twion wheels (Alber, Albstadt, Germany). These wheels contain a sensor in the handrim and an electric motor (60 W, 20 Nm) that delivers power proportionally to the input of the wheelchair user. The wheels were mounted on a Küschall k-series wheelchair (Witterswil, Switzerland). In the RHW mode conventional 24-in. wheels were used on the same wheelchair. Tire pressure was checked before each session by the same researcher and kept constant at six bars (600 kPa). Anti-tip wheels were mounted to prevent backward falls.

### (Assisted-) mechanical efficiency & energy expenditure

External power output was approximated with a drag test as described by Van der Woude and colleagues [9]. A weight on a pulley was attached to the wheelchair to ensure all participants performed at 0.21 W/kg bodyweight external power output in both groups [31]. The added power output was calculated by adding the acting gravity force of the pulley weight to the force measured by the drag test multiplied by the speed of the treadmill.

Breath-by-breath open circuit spirometry was performed with a Quark CPET (COSMED, Rome, Italy). The internal energy expenditure (EE), assumed to be generated by glucose and lipid oxidation, and lipogenesis (W), was determined with the equation of Garby and Astrup [32].

(Assisted-) mechanical efficiency was derived from the ratio between the external power output (W) and the metabolic energy used internally during steady-state submaximal exercise and expressed as a percentage.

### Heart rate & perceived exertion

Heart rate was also measured by the Quark CPET with a sensor placed across the chest area and a wireless connection. Additionally, participants were asked to give their physical exertion a score between 6 and 20 on the Borg rate of perceived exertion (RPE) scale [33].

### Control

An active-marker Optotrak system (NDI, Waterloo, Canada) was used to detect two cluster markers. Position data were sampled at 100 Hz with two Optotrak modules, each containing three cameras. The first cluster marker was placed on the wheelchair to determine the position of the wheelchair with respect to the treadmill. The position of the wheelchair was represented by the center of the rear axles. This was calculated by creating virtual markers on the right and left axle of the wheel in relation to the wheelchair cluster and averaging their position. Control was defined as the standard deviation in the right-left direction and the forward-backward direction of the wheelchair position. Additionally, push frequency was determined from the position of the second cluster marker, which was placed on the right hand of the subject.

### Statistical analysis

All data were first processed and cut in MATLAB (Mathworks, Natick, USA) using custom-written scripts. Only the last minute of each four-minute block was used for analysis. The three separate four-minute blocks per session were averaged. Subsequently, outcome variables were exported to IBM SPSS version 23 (SPSS Inc., Chicago, USA). The level for statistical significance was set at 0.05 for all tests. All data were checked for deviations from a normal distribution. To evaluate the effect of practice, a mixed analysis of variance (ANOVA) was used with group and time as independent variables. Data on (assisted) mechanical efficiency, heart rate, RPE, push frequency, and treadmill position were compared for time and for group effects. If an interaction effect was found, a separate repeated-measures ANOVA was conducted for each group. This allowed for the analysis of time effects for each group separately. Finally, a MANOVA was used to check for between-group effects. An independent t-test with Bonferroni correction was used to compare T1 of one group with the transfer session of the other group.

## Results

**Fig. 3:**
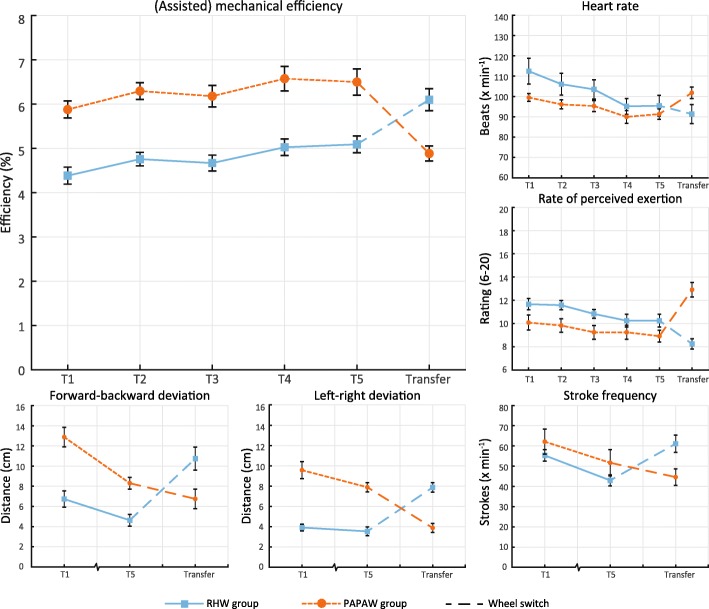
Average (assisted) mechanical efficiency, heart rate, RPE, left-right and forward-backward deviation, and stroke frequency of each session for the RHW group (blue, *n* = 12) and the PAPAW group (orange/dotted, n = 12). Standard error bars are given for each session

No differences were found in the characteristics of the participants (Table 1). All participants completed the protocol at a mean external power output of 17 W, though some safety stops were necessary to ensure their safety in the initial bouts, this predominantly happened in the PAPAW group during T1 in the first minute (x̅RHW = 0.30 stops, x̅PAPAW = 3.25 stops). No emergency stops were needed after T1 for the RHW group and after T4 for the PAPAW group. During the transfer of the RHW group to the PAPAW mode some emergency stops were needed (x̅=0.92 stops). There were no adverse incidents resulting in injury. If a safety stop was made the protocol was resumed from that point on. Kinematic data were missing on three occasions and could not be included in the analysis. Results (Fig. 3, Table 2) and statistical outcomes (Table 3) for (assisted) mechanical efficiency, heart rate, RPE, stroke frequency and position on the treadmill will be discussed below.

**Table 2:** Descriptive statistics (mean ± sd) for (assisted) mechanical efficiency (ME), energy expenditure (EE), heart rate (HR), rate of perceived exertion (RPE), stroke frequency (stroke), left-right (Dev L-R) and forward-backward (Dev F-B) deviation in the RHW group (*n* = 12) and the PAPAW group (*n* = 12)

Variable	Group	T1	T2	T3	T4	T5	Transfer
ME (%)	RHW	4.38 (0.67)	4.76 (0.53)	4.67 (0.62)	5.02 (0.63)	5.09 (0.66)	6.10 (0.85)
	PAPAW	5.88 (0.67)	6.29 (0.66)	6.18 (0.85)	6.57 (0.96)	6.50 (1.02)	4.88 (0.59)
*EE (W)*	RHW	380 (75.1)	349 (73.8)	353 (53.4)	329 (58.6)	323 (59.0)	272 (51.8)
	PAPAW	285 (48.5)	265 (41.1)	271 (42.1)	255 (44.9)	255 (45.0)	345 (67.0)
*HR (bpm)*	RHW	112 (22.0)	106 (18.7)	104 (16.4)	95.1 (13.5)	95.5 (17.8)	91.3 (16.0)
	PAPAW	99.5 (6.70)	96.1 (7.73)	92.3 (9.50)	90.0 (10.9)	91.3 (8.99)	102 (9.95)
*RPE (6–20)*	RHW	11.7 (1.67)	11.6 (1.38)	10.8 (1.34)	10.3 (1.91)	10.3 (1.91)	8.25 (1.54)
	PAPAW	10.1 (2.23)	9.83 (2.04)	9.25 (2.01)	9.25 (2.10)	8.92 (1.78)	12.9 (2.15)
*Stroke (n x min* ^*−1*^ *)*	RHW	55.3 (9.90)				43.0 (9.30)	61.1 (14.3)
	PAPAW	62.1 (21.6)				51.7 (21.7)	44.6 (12.9)
*Dev F-B (m × 10* ^*−2*^ *)*	RHW	6.73 (2.82)				4.63 (2.03)	10.7 (3.78)
	PAPAW	12.9 (3.33)				8.30 (1.94)	6.75 (3.03)
*Dev L-R (m ×10* ^*−2*^ *)*	RHW	3.91 (1.12)				3.54 (1.49)	7.87 (1.56)
	PAPAW	9.57 (2.92)				7.90 (1.51)	3.88 (1.42)

**Table 3:** Statistical outcomes for (assisted) mechanical efficiency (ME), energy expenditure (EE), heart rate (HR), rate of perceived exertion (RPE), stroke frequency (stroke), left-right (Dev L-R) and forward-backward (Dev F-B) deviation in the RHW group (*n*=12) and the PAPAW group (*n*=12)

Variable	Group	Interaction-effect		Group-effect		Time-effect		Pre-post^b^	Transfer^f^
		F(df)	*p*	F(df)	*p*	F(df)	*p*	*p*	*p*
ME (%)	RHW	0.07(4,88)	0.990	39.1(1,22)	*<0.001*	7.26(4,88)	*<0.001*	*0.003*	0.242
	PAPAW								0.032
*EE (W)*	RHW	1.44(4,88)	0.227	15.7(1,22)	*0.001*	8.62(4,88)	*<0.001*	*0.001*	0.278
	PAPAW								0.125
*HR* ^*a*^ *(bpm)*	RHW	2.60(4,88)	*0.041*	1.16(5,18)	0.368^c^	14.6(4,44)	*<0.001*	*<0.001*	0.063^d^
	PAPAW					4.24(4,44)	*0.005*	*0.022*	0.071^d^
*RPE (6-20)*	RHW	0.45(4,88)	0.770	4.92(1,22)	*0.037*	7.02(3,59)	*0.001*	*0.032*	0.023^e^
	PAPAW								0.076^e^
*Stroke (n x min* ^*-1*^ *)*	RHW	0.17(1,22)	0.687	1.38(1,22)	0.253	32.1(1,21)	*<0.001*	*<0.001*	0.448
	PAPAW								0.020
*Dev F-B* ^*a*^ *(m x 10* ^*-2*^ *)*	RHW	6.73(1,22)	*0.017*	11.6(1,23)	*<0.001* ^*c*^	10.3(1,11)	*0.008*	*0.008*	0.082
	PAPAW					26.2(1,11)	*<0.001*	*<0.001*	0.495
*Dev L-R (m x10* ^*-2*^ *)*	RHW	3.67(1,22)	0.068	40.4(1,22)	*<0.001*	7.04(1,22)	*0.015*	*0.042*	0.048^d^
	PAPAW								0.474

Significant results are italicized (α=0.05)

^a^Separate repeated-measures ANOVAs were performed for each group

^b^Tested with simple contrasts

^c^Tested with a MANOVA with follow up ANOVAs

^d^Equal variances not-assumed

^e^Mann-Whitney U

^f^One-sided p-value (α=0.004)

### (Assisted-) mechanical efficiency & energy expenditure (T1-T5)

(Assisted) mechanical efficiency significantly increased over time (Table 2, Fig. 3) for both groups (ΔRHW.T1-T5 = 0.71%, ΔPAPAW.T1-T5 = 0.62%). A post-hoc analysis with simple contrasts showed that T1 significantly differed from T5 in both groups. Also, a between group difference was found with a higher mechanical efficiency for the PAPAW group compared to RHW (Δ = 1.21% at T5), however no significant interaction effect was found.

Energy expenditure (EE) is closely tied to mechanical efficiency as external power output was standardized. A significant reduction in EE was observed over time for both groups (ΔRHW.T1-T5 = -57 W, ΔPAPAW.T1-T5 = -30 W), this was corroborated by a post-hoc analysis with simple contrasts between T1 and T5. Additionally, EE was significantly lower in the PAPAW group than in the RHW group (Δ = 68 W at T5). No interaction effect for EE was found.

### Heart rate & perceived exertion (T1-T5)

An interaction effect between time and group was found for heart rate, where heart rate reduced more over time for the RHW group than for the PAPAW group (ΔRHW.T1-T5 = 16.5 bpm, ΔPAPAW.T1-T5 = 8.2 bpm). Therefore, a separate repeated-measures ANOVA for each group was conducted so time effects could be isolated for individual groups. Heart rate significantly reduced over time in the RHW group and in the PAPAW group. Post-hoc analysis with simple contrasts revealed that T1 significantly differed from T5 in both groups. A separate between-group analysis was conducted with a MANOVA and did not reveal a significant difference between groups.

Participants rated their physical exertion significantly lower over time (ΔRHW.T1-T5 = 1.4, ΔPAPAW.T1-T5 = 1.1). Moreover, the PAPAW group had a significantly lower RPE than the RHW group (Δ = 1.4 point on T5). Post-hoc analyses with simple contrasts revealed that participants scored significantly lower on RPE during T1 when compared with T5 and no interaction effects were found for RPE.

### Control (T1-T5)

Deviation of the forward-backward position on the treadmill contained an interaction effect as the PAPAW group showed a greater reduction over time, therefore, separate repeated-measures ANOVAs were used to compare the effect of time. It was found that the forward-backward deviation significantly reduced over time for the PAPAW group and for the RHW (ΔRHW.T1-T5 = 1.4, ΔPAPAW.T1-T5 = 1.1). Furthermore, a MANOVA, with follow-up ANOVAs revealed that the forward-backward deviation was significantly higher for the PAPAW group than the RHW group during T1 and T5.

Left-right deviation of the treadmill position significantly lowered between T1 and T5 for both groups. The PAPAW group had a significantly higher left-right deviation than the RHW group and no interaction effect was found.

Stroke frequency significantly lowered over time for both groups (ΔRHW.T1-T5 = 12.5, ΔPAPAW.T1-T5 = 10.4). Additionally, stroke frequency was found to be lower in the RHW group (Δ = 8.7 strokes x min^− 1^ at T5), however this difference was not statistically significant. No interaction effects were found.

### Transfer

Mechanical efficiency was higher for the PAPAW group during their transfer session than for the RHW group during their first session (Δ = 0.5%), however, these differences were not statistically significant. Similar results were found for EE (Δ = -35 W), heart rate (Δ = − 10 bpm), RPE (Δ = − 1.2), and stroke frequency (Δ = − 10.7 strokes x min-1), but these were also not significant.

Additionally, the RHW group had a lower heart rate (Δ = − 7.9 bpm), RPE (Δ = − 1.9), left-right (Δ = − 2.2 m × 10^− 2^) and forward-backward (Δ = − 1.7 m × 10^− 2^) deviation during their transfer session than the PAPAW group during their first session, however, none of these results reached statistical significance.

## Discussion

The aim of the current study was to explore early skill acquisition through practice of able-bodied participants in PAPAWs and compare them to able-bodied participants that performed the same protocol with RHWs. Learning effects were found for both groups, as both increased their (assisted) mechanical efficiency, reduced their heart rate, RPE, their right-left and forward-backward deviation on the treadmill, and had a lower stroke frequency over time.

Assisted mechanical efficiency in the PAPAW increased from 5.8 to 6.5%. These values are lower compared to those of Arva [16] and colleagues (9.9–20.6% at different speeds/power outputs), but higher than the findings of Pavlidou [34] and colleagues (4.2%). (Assisted) mechanical efficiency rises as external power output increases [16], which might explain the differences between our study and the study of Pavlidou and colleagues. However, Arva and colleagues had participants perform at a lower power output and still found a higher mechanical efficiency. Of course, the use of different amounts of power assist from the different PAPAWs could be the cause of this. For future studies, it seems important to consider that PAPAW propulsion, in addition to RHW propulsion, changes through learning.

Mean mechanical efficiency in the RHW mode was found to be slightly lower (5.1%) than in other studies (5.5–7.0%) with able-bodied subjects [21, 27, 35] and also increased over time. One possible explanation is that the way external power output is determined between different studies influences the outcomes. When external power output is determined from measurement wheels the effect of movement variability on the treadmill can also be included, increasing the observed external power output. In the current study the power output was determined from a drag-test, which determines the power output required for moving in a straight line at a constant velocity. In a paper of Vegter et al. where two measurement wheels and the drag test were compared an underestimation of 14% was found [20].

In general, PAPAW propulsion was more efficient than RHW propulsion. As external power output was constant during this study, a higher mechanical efficiency also corresponds with a reduction in EE. The lower EE [7, 13, 15, 16, 34, 36] is in accordance with other studies that compared RHWs with PAPAWs. Likewise, RPE was found to be lower for the PAPAW group and similar results were found in other studies [7, 11, 14]. Correspondingly, heart rate was also expected to be lower for the PAPAW group. Mean heart rates were in the expected direction, but due to the high variance between subjects no statistically significant difference could be found, which is in contrast with previous studies [11–13, 15, 34].

Unfortunately, it is not clear what proportion of the reduced energetic cost of PAPAW propulsion in comparison with RHW propulsion is due to the contribution of the motor. For example, it could be that the technique of the participant is less efficient, but the added power of the motor is enough to compensate for that. The assist provided by the wheels is of proportional grade with no exact knowledge of the algorithm of the assist. The assist being proportional means that the torque provided by the motors during propulsion is dependent on the propulsion characteristics of the user. Knowledge on the algorithm of the assist and the propulsion characteristics of the user are of great importance to objectively determine whether the mechanical efficiency is high or low compared to other studies. The motor power is rated at 60 W per wheel, considering the low intensity required for this task (17 W, fixed speed and direction) it can be stated that the motors do not use their full potential. Although it is good to see that the user needs to stay somewhat active. Different task constraints might give different results due to their interaction with the control algorithm.

The control parameters, left-right and forward-backward deviation, were also found to be higher in the PAPAW group than in the RHW group. This was in line with the expectation that the PAPAW would be harder to control, especially considering the proportional control of the power assist wheels. This finding is in agreement with other authors that have already stated that a PAPAW may be helpful for specific tasks that require more torque, but the PAPAW is less suited for tasks that require a high amount of control [10, 37]. However, user control did improve over time, indicating that some practice is needed to properly use a PAPAW and evaluate the impact of a PAPAW. Clinicians and dealerships should therefore consider a trial period for potential new users. As the factor of control is not often used in research it is still unclear how different PAPAWs vary in their controllability and what this effect means for the user in terms of efficiency and ease of use during activities of daily living. Standardized measures like the one proposed in this study could be used to quantify the control over the wheelchair. However, to be able to generalize results to real life propulsion the task might need to be expanded (e.g. with obstacle avoidance).

Stroke frequency reduced over time in both groups and the PAPAW group used a higher stroke frequency than the RHW group on average [17], however, this difference was not significant. Ideally, the PAPAW would require fewer pushes to maintain the same velocity as the motors continue to engage for a short period of time after release. A decrease in stroke frequency over time could show that less adjustments have to be made to maintain a steady position on the treadmill. Moreover, it could also explain why mechanical efficiency in this study was lower than in other studies for the PAPAW, as other studies have also shown that an increase in movement amplitude and a decrease of movement frequency reduces the energy cost of a task [23, 24, 38, 39]. The lack of control in the PAPAW group is the most likely explanation for the higher stroke frequency. Maintaining a straight line on the treadmill, when not in control, urges the user to apply corrections during propulsion resulting in a higher stroke frequency. This study did not evaluate whether these pushes were unilateral (correction) or bilateral. In this experiment, the wheelchair was not attached to the treadmill with a rail or a fixation system. In experiments where such a system is used the factor of control becomes less important, this might give an unfair advantage to PAPAWs in some comparison studies.

As PAPAW propulsion is similar to regular handrim wheelchair propulsion, a skill transfer between these two modes was hypothesized to exist [40]. From a learning perspective, a positive influence on mechanical efficiency might have advocated the use of PAPAWs until a sufficient level of skill is reached. A positive influence in control could have pointed towards the use of RHWs in early practice in order to gain sufficient control before transferring to the more demanding (in terms of control) PAPAW mode. Tendencies of a positive transfer effect were observed in the data, though no statistically significant effects could be found. For now, based on the zero transfer, it is recommended that participants practice or receive training in a new mode before choosing to settle with a configuration. Transfer tendencies were also observed for RPE, where prior experience in a RHW or PAPAW led to a non-significant (after Bonferroni correction) difference in perceived exertion. Even though no statistically significant difference was found, researchers and clinicians should still beware of changes in perception due to prior experience or a response bias.

It should also be noted that there were some limitations in this study. Although significant between-group differences for left-right and forward-backward deviation were already found, the values for left-right and forward-backward deviation are conservative for the PAPAW group, with an average of three emergency stops during the first session. The data during an emergency stop was not used, but an emergency stop shows a high amount of deviation and a high stroke frequency, this would have influenced the outcome for the PAPAW group during the first session. During the transfer session of the RHW group there were less emergency stops. It may be that the RHW group was more skillful through their previous experience, but the statistical analysis failed to support this finding.

Moreover, the control task could have been unclear for some participants. We instructed our participants to ‘stay in the center of the treadmill as best as they could’. A projected target on the treadmill could have helped participants in staying in the center of the belt. These targets can then also be used to produce a more generalizable task (e.g. with obstacle avoidance). Furthermore, large individual differences between participants were observed. Recent research has shown that individual motor learning differences are important to take into account in motor learning studies [21, 27]. In this study, it appeared that some participants were able to capitalize more on the benefits of a PAPAW than other participants, as indicated by the high variability between participants. However, with the current sample size and study design it was not possible to analyze these individual differences.

Finally, mechanical efficiency, RPE, stroke frequency, and right-left deviation did not show any interaction effects, indicating that learning rate between the two modes might be similar. The difference in difficulty between the two modes could have led to differences in motor learning strategies. However, without knowledge of the kinetics of the user and the algorithm of the PAPAW we cannot make inferences about the underlying motor learning principles of (assisted) wheelchair propulsion. Future research into the kinetics and kinematics of PAPAW propulsion is needed to provide more insight in the adaptations that are made during the learning process.

## Conclusions

The present study showed that the energetic cost of regular handrim and power-assisted wheelchair propulsion can be lowered through low-intensity practice in users with no previous experience. Power-assisted wheelchair propulsion requires less energy than regular handrim wheelchair propulsion, but power-assist wheels are more difficult to control. Based on these results, users should be able to practice with a power-assisted wheelchair before evaluating whether the user can benefit from the technology. Additionally, a tendency was found indicating that the use of subjective scales for exertion might be influenced by the previous experience of the user, which should be taken in consideration when presenting a power-assist wheel to a new user. Additional research on the effects of practice with assistive technology is needed to improve efficient use and design. More knowledge about effective power-assist algorithms is needed, especially with respect to the control needs of the user. These studies would benefit from kinetic data measured at the handrim and information on the power-assist algorithms.

## Abbreviations

ANOVA: Analysis of variance
EE: Energy expenditure
PAPAW: Pushrim-activated power-assisted wheelchair
RHW: Regular handrim wheelchair
RPE: Rate of perceived exertion
